# Hypertrophic cardiomyopathy clinical phenotype is independent of gene mutation and mutation dosage

**DOI:** 10.1371/journal.pone.0187948

**Published:** 2017-11-09

**Authors:** Shiv Kumar Viswanathan, Heather K. Sanders, James W. McNamara, Aravindakshan Jagadeesan, Arshad Jahangir, A. Jamil Tajik, Sakthivel Sadayappan

**Affiliations:** 1 Heart Lung Vascular Institute, Division of Cardiology, Department of Internal Medicine, University of Cincinnati, Cincinnati, Ohio, United States of America; 2 Department of Cell and Molecular Physiology, Center for Translational Research and Education, Health Sciences Division, Loyola University Chicago, Maywood, Illinois, United States of America; 3 Aurora Cardiovascular Services, St. Luke’s Medical Center, Milwaukee, Wisconsin, United States of America; 4 Center for Integrative Research on Cardiovascular Aging (CIRCA), Aurora Health Care, Milwaukee, Wisconsin, United States of America; Indiana University, UNITED STATES

## Abstract

Over 1,500 gene mutations are known to cause hypertrophic cardiomyopathy (HCM). Previous studies suggest that cardiac β-myosin heavy chain (*MYH7)* gene mutations are commonly associated with a more severe phenotype, compared to cardiac myosin binding protein-C (*MYBPC3)* gene mutations with milder phenotype, incomplete penetrance and later age of onset. Compound mutations can worsen the phenotype. This study aimed to validate these comparative differences in a large cohort of individuals and families with HCM. We performed genome-phenome correlation among 80 symptomatic HCM patients, 35 asymptomatic carriers and 35 non-carriers, using an 18-gene clinical diagnostic HCM panel. A total of 125 mutations were identified in 14 genes. *MYBPC3* and *MYH7* mutations contributed to 50.0% and 24.4% of the HCM patients, respectively, suggesting that *MYBPC3* mutations were the most frequent cause of HCM in our cohort. Double mutations were found in only nine HCM patients (7.8%) who were phenotypically indistinguishable from single-mutation carriers. Comparisons of clinical parameters of *MYBPC3* and *MYH7* mutants were not statistically significant, but asymptomatic carriers had high left ventricular ejection fraction and diastolic dysfunction when compared to non-carriers. The presence of double mutations increases the risk for symptomatic HCM with no change in severity, as determined in this study subset. The pathologic effects of *MYBPC3* and *MYH7* were found to be independent of gene mutation location. Furthermore, HCM pathology is independent of protein domain disruption in both *MYBPC3* and *MYH7*. These data provide evidence that *MYBPC3* mutations constitute the preeminent cause of HCM and that they are phenotypically indistinguishable from HCM caused by *MYH7* mutations.

## Introduction

Hypertrophic cardiomyopathy (HCM) is a commonly inherited myocardial disease with an estimated prevalence of 1 in 200 [1]. HCM is characterized by abnormal thickening of the left ventricular wall that cannot be explained by abnormal loading conditions or metabolic disorder [2]. In patients with HCM, cardiomyocytes enlarge, and interstitial fibrosis and myocardial disarray are apparent [3]. Clinical outcomes of HCM are highly variable such that many patients are asymptomatic, or mildly symptomatic, presenting with symptoms such as dyspnea, angina, syncope, lightheadedness, and heart palpitations [2]. In severe cases, HCM is potentially life- threatening and associated with high risk of heart failure, atrial fibrillation, stroke, and sudden death [4]. Importantly, HCM is the leading cause of sudden cardiac death in young athletes [5]. However, even among HCM, it is difficult to predict disease severity owing to the variable penetrance of the disease and insufficient genotype-phenotype correlation [6].

Genetically linked HCM has been defined mostly as a disease of the sarcomere [7]. Mutations in genes encoding sarcomeric proteins account for over 95% of all HCM cases. Over 1,500 autosomal dominant mutations in at least 14 genes encoding cardiac sarcomere proteins are associated with HCM [7–10]. Mutations in cardiac β-myosin heavy chain (*MYH7*) and cardiac myosin binding protein-C (*MYBPC3*) occur in most cases [11, 12], while cardiac troponins -I (*TNNI3*),–T (*TNNT2*), and–C (*TNNC1*), α-tropomyosin (*TPM1*) and the myosin light chains *(MYL2* and *MYL3*) are less frequently associated with HCM. These mutations display a wide range of phenotypes, from normal appearing heart, or mild hypertrophy, to severe hypertrophy and increased risk for life-threatening ventricular arrhythmias [1, 13, 14]. In patients with HCM, new mutations in sarcomeric proteins are increasingly recognized, but genotype-phenotype correlation and the underlying molecular mechanisms of HCM pathogenesis have not been fully defined. Two lines of reasoning have been advanced to explain the pathogenic effects of *MYH7* and *MYBPC3* mutations. In some studies, *MYBPC3* mutants have been associated with mild to moderate phenotype and a less severe form of HCM [15, 16] with late clinical onset [17, 18], while other studies have described *MYBPC3* mutations to be associated with more severe phenotype [19–22]. Furthermore, some studies have demonstrated that HCM patients with multiple causative gene mutations have poorer prognosis in terms of earlier disease onset, increased LV hypertrophy, and increased frequency of heart failure and sudden cardiac death, when compared to those carrying a single mutation [11, 23–26]. Studies have also reported that 70% of the mutations in *MYBPC3* are truncations and cause a more severe HCM phenotype than that associated with missense and in-frame deletions [27, 28].

Therefore, the primary purpose of this study was to further define the spectrum of mutations in a cohort of patients examined in a specialized center for HCM and define the effect of compound mutations on clinical pathogenesis. We also asked if individual gene mutations and the protein domains they affect could be correlated with the severity of HCM. Our data demonstrate that *MYBPC3* gene mutations are the predominant cause of HCM with high penetrance. Additionally, the severity of HCM was shown to be dependent on the predicted pathogenicity of the mutation, irrespective of the gene (*MYBPC3* and *MYH7*), protein domain affected, or compound effects.

## Results

### Hypertrophic cardiomyopathy cohort characteristics

**Fig 1:**
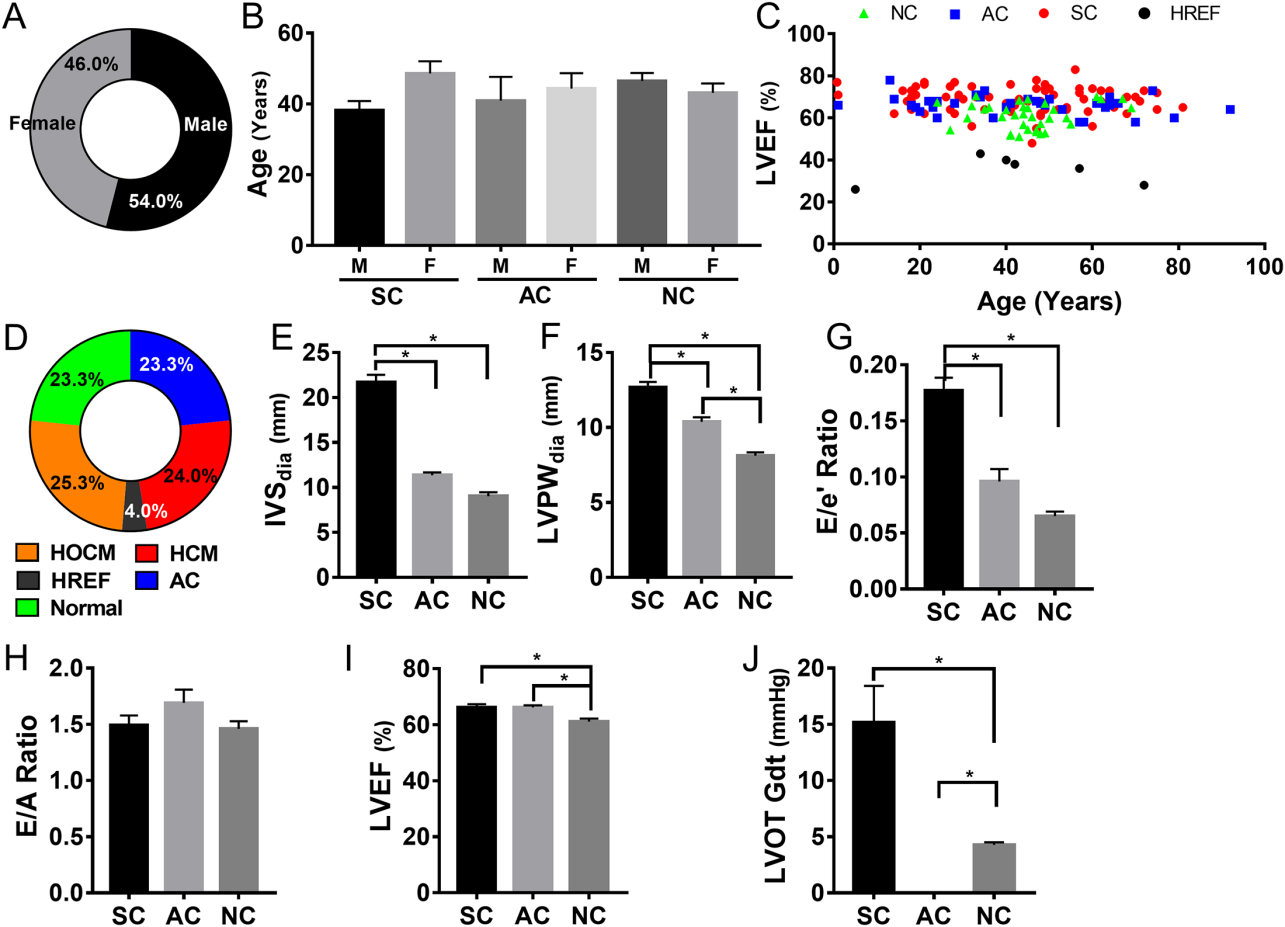
Study subject characteristics across three cohorts and clinical evaluation profile. **A**, Distribution of males and females in the study population represented as percentages. **B**, Age and gender distribution among the three cohorts, probands with definitive HCM/HOCM or dHCM clinical phenotype (symptomatic carriers, SC), family members with no HCM phenotype (asymptomatic carriers, AC), and non-carriers (NC) unrelated community-based population confirmed by echocardiography to have normal cardiac structure and function. Data are represented as means with standard error of mean (SEM) **C**, Age-based distribution of left ventricular ejection fraction across the three cohorts; red markers indicate HCM, blue markers indicate AC, and green markers indicate NC. Black markers indicate dHCM subjects with ejection fraction ≤45%. **D**, Diagnostic subclassification of subjects with gene mutation represented as a percentage of total. **E-J** panels show various echocardiographic parameters that reflect HCM severity presented as mean with standard error of mean. IVS, interventricular septum thickness (**E**); LVPW, left ventricular posterior wall (free wall) thickness (**F**); E/e’ ratio, ratio of early transmitral flow (E) to left ventricular early diastolic velocity (e’) (**G**); E/A ratio, ratio of early (E) to late (A) ventricular filling velocities (**H**); LVEF, left ventricular ejection fraction (**I**); LVOT-Gdt, LV outflow tract peak gradient (**J**). * indicates p value of 0.01 or lower by one-way ANOVA analysis.

One hundred and fifty consenting participants were enrolled in this study. The study group consisted of 81 (54.0%) males and 69 (46.0%) females (Fig 1A) with an average age of 42.9±17.3 and 45.9±18.3 years, respectively. The overall age distribution between the groups was comparable with no significant bias in any group (Fig 1B and Table 1). The study population was uniformly distributed in terms of age and left ventricular (LV) function with the exception of six patients who presented with HCM that had progressed to the decompensated stage (dHCM) (Fig 1C, black marks). The clinical characteristics of the study population, as evaluated on the basis of American Heart Association (AHA) clinical guidelines, showed that 24.0% (n = 36) had HCM without obstruction, while 25.3% (n = 38) had hypertrophic obstructive cardiomyopathy (HOCM) (Fig 1D). Interestingly, concentric hypertrophy (hypertension with IVS>10mm and IVS/PWT ≤1.1) was identified in 4.7% (n = 7), but only among asymptomatic carriers and not within non-carriers (data not shown). Hypertension and diabetes were observed in 38.0% (n = 57) and 7.3% (n = 11) of subjects, respectively (Table 1). Electrocardiographic assessment of patients and first-degree family members showed no significant ECG abnormalities in 55 (36.7%), paced rhythm in 14 (9.3%), and bundle branch block in 10 (6.7%) patients (6 with right and 4 with left bundle branch block) (Table 2).

**Table 1:** Study subject characteristics across three cohorts.

	Symptomatic Carriers (SC)			Asymptomatic Carriers (AC)			Non-Carriers (NC)			p value
	Mean ± SEM	Min	Max	Mean ± SEM	Min	Max	Mean ± SEM	Min	Max	
Number of Subjects	80			35			35			
Percentage	53.3%			23.3%			23.3%			
Mean Age (yrs)	42.2 ± 2.1			43.3 ± 3.6			45.1 ± 1.7			
Males	50			11			20			
Females	30			24			15			
Hypertension (% Positive)	43%			26%			20%			
Diabetes (% Positive)	9%			9%			3%			
LVEF (%)	66.2 ± 1.2	26.0	83.0	66.2 ± 0.8	58.0	78.0	61.2 ± 1.0	51.1	70.5	<0.05*†
IVS (cm)	21.7 ± 0.8	6.0	44.0	11.4 ± 0.3	7.0	14.0	9.0 ± 0.4	5.0	15.0	<0.05*‡
LVPW (cm)	12.7 ± 0.4	7.0	25.0	10.4 ± 0.3	8.0	14.0	8.1 ± 0.2	6.0	11.0	<0.05†*‡
LVOT Gradient (mmHg)	15.2 ± 3.3	0	160.0	0	0	0	4.3 ± 0.2	2.1	9.6	<0.05*‡
E/A Ratio	1.5 ± 0.1	0.6	4.6	1.7 ± 0.1	0.8	3.9	1.5 ± 0.1	0.9	2.3	n.s
E/e' Ratio	17.7 ± 1.1	3.1	71.4	9.6 ± 1.1	4.0	31.0	6.5 ± 0.4	4.0	16.0	<0.05*‡

Echocardiography measurements were taken under normal and conscious conditions. LVEF, left ventricular ejection fraction; IVS, interventricular septal thickness; LVPW, left ventricular posterior wall thickness; LVOT Gradient, LV outflow track E/A Ratio, ratio of early transmitral flow (E) to late flow owing to atrial contraction (A); E/e’ Ratio, ratio of early transmitral flow (E) to left ventricular early diastolic velocity (e’). Statistical significance was calculated using one-way ANOVA; symbols used in p value comparison

* SC *vs*. NC;

^†^ AC *vs*. NC;

^‡^ SC *vs*. AC.

**Table 2:** Distribution of ECG findings across the four different phenotypic groups.

Diagnosis	Normal Rhythm	Paced Rhythm	AF	LBBB	RBBB	R/LVH	LAE	Septal Reduction
HCM	12	6	1	1	3	8	5	
HOCM	12	6		2	1	9	10	4
dHCM	4	1		1	1		1	1
AC	27	1		0	1	3		
Total	55	14	1	4	6	20	16	5

ECG abnormalities include presence of paced rhythm from implanted pacemaker/ICD, atrial fibrillation (AF), left or right bundle branch block (LBBB, RBBB) and right or left ventricular hypertrophy (R/LVH) and left atrial enlargement (LAE). Diagnosis types include hypertrophic cardiomyopathy (HCM), hypertrophic obstructive cardiomyopathy (HOCM), defined as LVOT gradient ≥ 30mmHg at rest and an increase ≥ 50mmHg on provocation, and decompensated HCM (dHCM) characterized by an LVEF of ≤ 45%. AC; asymptomatic carriers.

Electrocardiographic analysis showed that the majority of subjects had normal sinus rhythm, while subjects with and without HCM phenotype showed some of the features commonly seen among HCM patients. These included ventricular hypertrophy (n = 20), bundle branch blocks (n = 10) and left atrial enlargement 16 (13.9%). Five subjects had previously undergone septal reduction, and 14 had either an implanted pacemaker or implantable cardioverter defibrillator (ICD) (Table 2). Phenotypically, septal and left ventricular posterior wall thickness and E/e’ ratio was significantly higher in HCM patients compared to both asymptomatic carriers and non-carriers (Fig 1D–1F, p<0.001). Interestingly, left ventricular ejection fraction in symptomatic carriers (68.4 ± 0.7%) was significantly higher than the non-carriers (61.2 ± 1.0%, p<0.014), and asymptomatic carriers (66.1 ± 0.9%, p<0.04) showed a significant increase in LVEF compared to the non-carriers (Fig 1I). The resting left ventricular outflow tract (LVOT) gradient was ≥30mmHg in 15 subjects (12.8%) and increased by ≥ 50mmHg with provocation in 14 (12.0%) patients (data not shown). Of the 55 familial samples screened, 38 (69.1%, probands and first-degree family members) were clinically found to have HCM/HOCM/dHCM, while 17 (30.9%) were asymptomatic carriers at the time of evaluation (S1 Table). The overall disease penetrance was 69.6% (80 of 115) of the subjects who were symptomatic carriers with HCM phenotype (S2 Table).

### Mutation profile of study cohort

**Fig 2:**
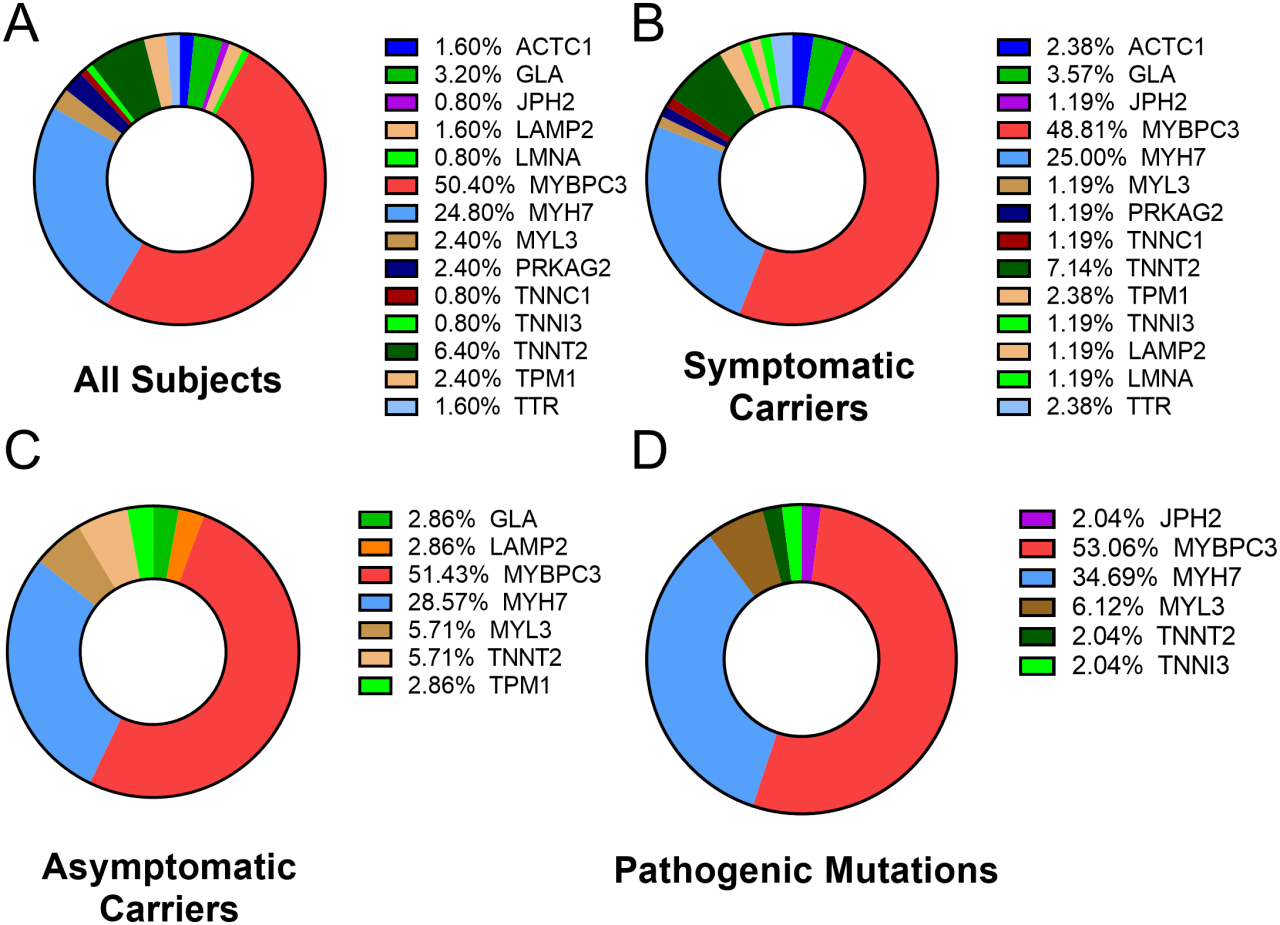
Genetic evaluation profile of the study cohort. **A-C**, show the percentage of gene mutation contribution among all subjects (**A**); HCM (**B**); and AC (**C**). Pathogenic mutation distribution among the various causative genes (**D**). *MYBPC3* and *MYH7* mutations are causative in >80% of all subjects, irrespective of their current phenotypic pathogenicity.

**Fig 3:**
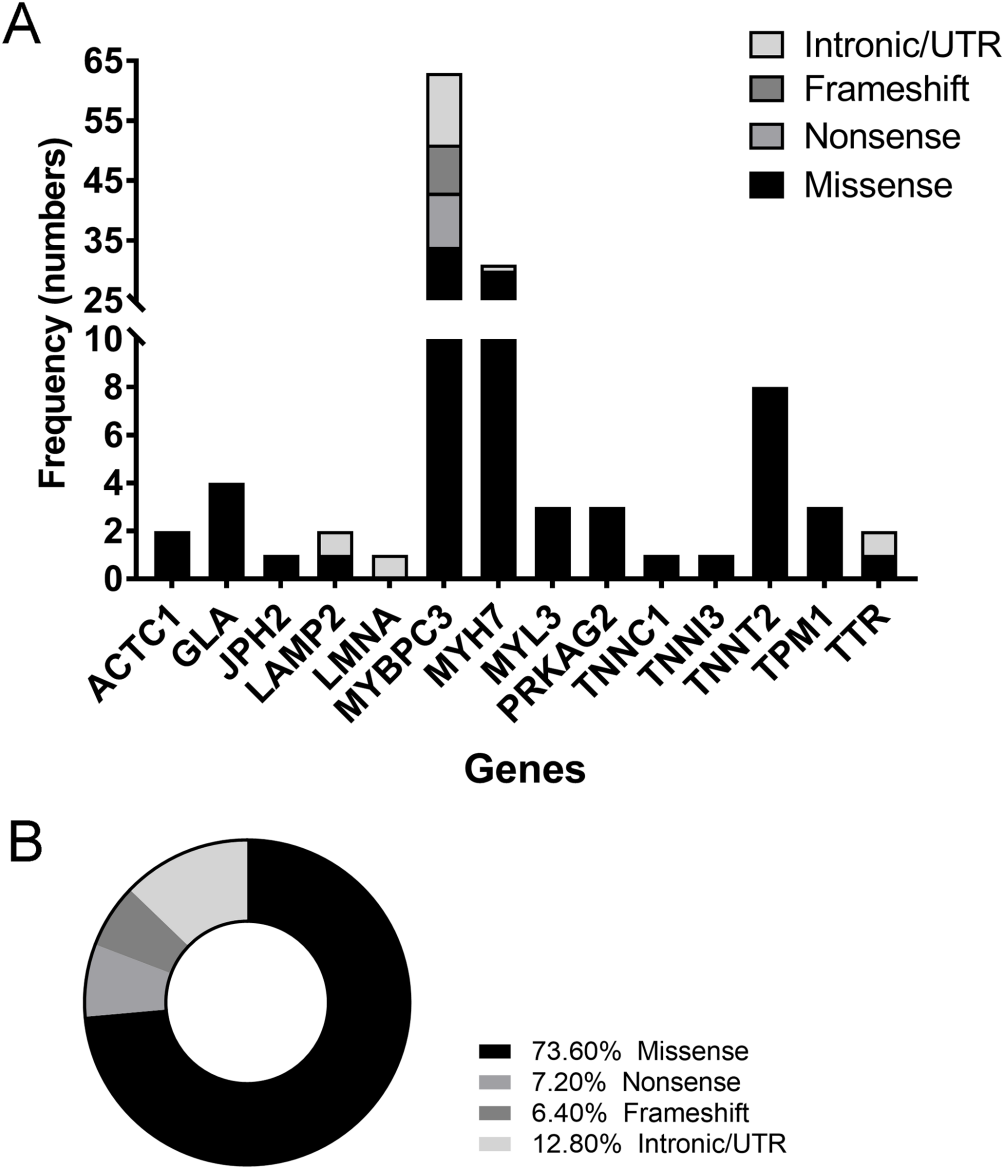
Frequency distribution of mutations in HCM-causing genes. The mutation distribution in 13 of the 18 HCM causative genes based on mutation class, including missense, nonsense, frameshift mutations and intronic and UTR variations (**A**). Distribution of mutation types among the causative genes (**B**), with missense and nonsense mutation contributing more than 80% of all classes of mutations.

Eighteen genes currently known to cause HCM were analyzed using next-generation sequencing (NGS) in these samples (S3 Table). Within the study cohort (n = 150), 39.3% had genetic variations in *MYBPC3*, and 20.7% had variations in *MYH7*, followed by cardiac troponin T (5.3%), α-galactosidase (2.7%), and tropomyosin and myosin light chain 3 (2% for both). One hundred twenty-five genetic mutations were found among the 18 genes, 82 of them being distinct changes. Of these distinct mutations, *MYBPC3* contributed 50.4%, while *MYH7* contributed 24.8% (Fig 2A). Of all potentially pathogenic mutations, including likely pathogenic, pathogenic and variations of conflicting pathogenicity, identified among the subjects (n = 115), it is significant to note that unique *MYBPC3* and *MYH7* contributed 50% and 24.4%, respectively, together accounting for about 74.4% of the total pathogenic mutational load (Fig 2D). These results suggest a very clean population of HCM subjects, asymptomatic carriers and unrelated non-carriers. Upon closer scrutiny, we identified all classes of genetic variations in the dataset with most (73.6%, n = 92) being missense, followed by nonsense (7.2%, n = 9) and frameshift mutations (6.4%, n = 8). A total of 12.8% (n = 16) had variations in intronic or the upstream regulatory region of the gene (Fig 3A and 3B). Seven of the 63 mutations in *MYBPC3* were novel, while 2 novel mutations were identified among the 31 *MYH7* mutations (Table 3).

**Table 3:** Novel mutations identified in *MYBPC3 and MYH7* in this study.

Gene	Nucleotide change	Protein modification	Mutation Type	Protein Region
***MYBPC3***	c.927-9G>A	IVS11-9G>A	Missense	M domain
	c.1028delC	p.Thr343MetfsX7	Frame Shift	M domain
	c.1235123delITT	p.Phe412Ter	Nonsense	C2 domain
	c.2455_2459delATGCG	p.Met819AlafsX12	Frame Shift	C6 domain
	c.2864_2865delCT	p.Pro955ArgfsX95	Frame Shift	C7 domain
	c.3192dupC	p.Lys1065GlnfsX12	Frame Shift	C8 domain
	c.3776delA	p.Gln1259ArgfsX72	Frame Shift	C10 domain
***MYH7***	c.563C>A	p.Thr188Asn	Missense	Motor
	c.4399C>G	p.Leu467Val	Missense	Motor

List of novel mutations and their effect on protein modification likely to be clinically relevant. The mutations are listed in the order of their location in the amino acid sequence, their functional consequence and the corresponding protein domain affected.

### *MYBPC3* mutations account for most genetically linked HCM

Disease penetrance with respect to individual genes was calculated to be 77.8% (n = 21) for *MYBPC3* and 71.4% (n = 10) for *MYH7*. A broader classification provided a penetrance of 78.4% (40 of 51) for sarcomeric genes and 66.7% (2 of 3) for non-sarcomeric genes (Table 4). Clinically, *MYBPC3* was associated with all major phenotypes, particularly HCM and HOCM, but also dHCM (S2 Table). These data show that *MYBPC3* mutations are the predominant cause of HCM in our patient cohort (n = 59), significantly higher than *MYH7* (n = 31), in contrast to previous reports [29, 30]. The remaining genetic contributions were similar to those already published, which showed that mutations from sarcomeric genes were far greater (~90%) than mutations from non-sarcomeric genes in less than 10% of the samples.

**Table 4:** Gene-wide distribution of HCM penetrance among familial samples.

	Sarcomeric Genes								Non-Sarcomeric Genes	
Genes	*ACTC1*	*MYBPC3*	*MYH7*	*MYL3*	*TNNC1*	*TNNI3*	*TNNT2*	*TPM1*	*GLA*	*LAMP2*
**Gene-positive**		**6**	**4**	**1**				**1**		**1**
**Phenotype- positive**	**2**	**21**	**10**	**1**	**1**	**1**	**3**	**1**	**2**	
**% Penetrance**	100*	**77.8**	**71.4**	50*	100*	100*	100*	50*	100*	0*
**% Penetrance**	**78.4**								66.7*	

Genes are classified in turn as either sarcomeric or non-sarcomeric.

*indicates penetrance values that might not reflect the true population-wide penetrance values owing to small sample size and low prevalence of the gene mutation.

### HCM phenotype is independent of underlying gene mutation

**Fig 4:**
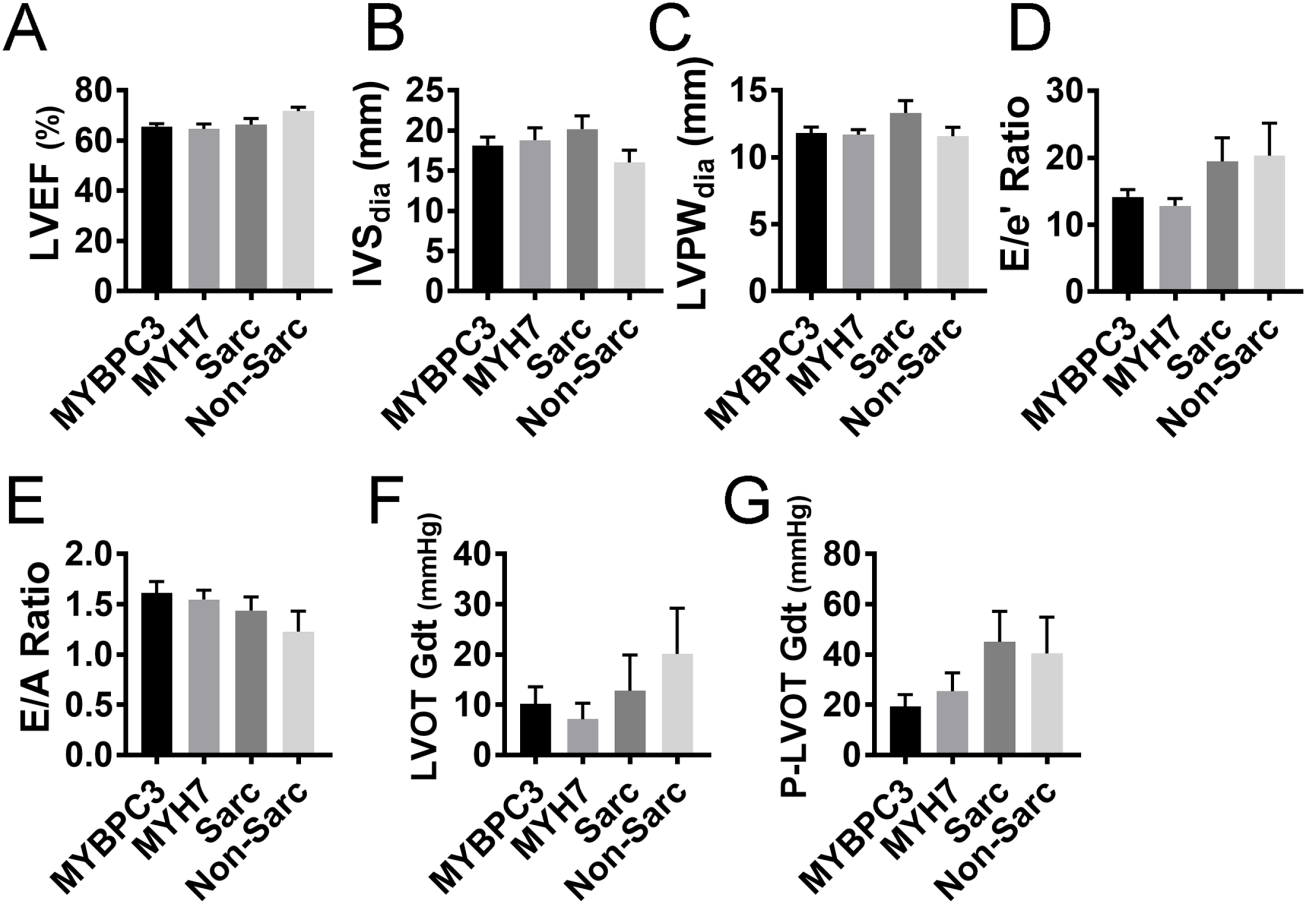
Phenotypic effect of mutations in *MYBPC3*, *MYH7*, sarcomeric (Sarc) and non-sarcomeric (Non-Sarc) genes. The severity of pathogenesis, as measured by echocardiography (**A-G)**. Echo parameters that reflect HCM severity include LVEF, left ventricular ejection fraction (**A**); IVS, interventricular septum thickness (**B**); LVPW, left ventricular posterior wall (free wall) thickness (**C**); E/e’ ratio, ratio of early transmitral flow (E) to left ventricular early diastolic velocity (e’) (**D**); E/A ratio, ratio of early (E) to late (A) ventricular filling velocities (**E**); LVOT-Gdt, LV outflow tract peak gradient (**F**) and provoked LVOT gradient (P-LVOT Gdt) (**G**). All parameters are represented as mean with SEM.

Since *MYH7* gene variations were previously reported as having a more severe phenotype compared to *MYBPC3* mutations, we compared phenotypic outcomes that resulted from variations in these two highly mutable genes. We found that *MYBPC3* (n = 59) and *MYH7* (n = 31) were still the genes most frequently associated with HCM. However, no significant phenotypic differences in symptomatic carriers were found between those caused by *MYBPC3* or *MYH7*, either by echocardiogram or by comparing other sarcomeric (n = 18) with non-sarcomeric gene mutations (n = 12) (Fig 4). We also compared symptomatic and asymptomatic carriers, and, again, no significant difference was found between the two genetic variants (data not shown). Taken together, these data show that the resultant HCM phenotype was independent of the offending mutation, even though *MYH7* and *MYBPC3* were the most dominant cause of HCM.

### Mutation dose increases the risk, but not severity, of HCM

**Fig 5:**
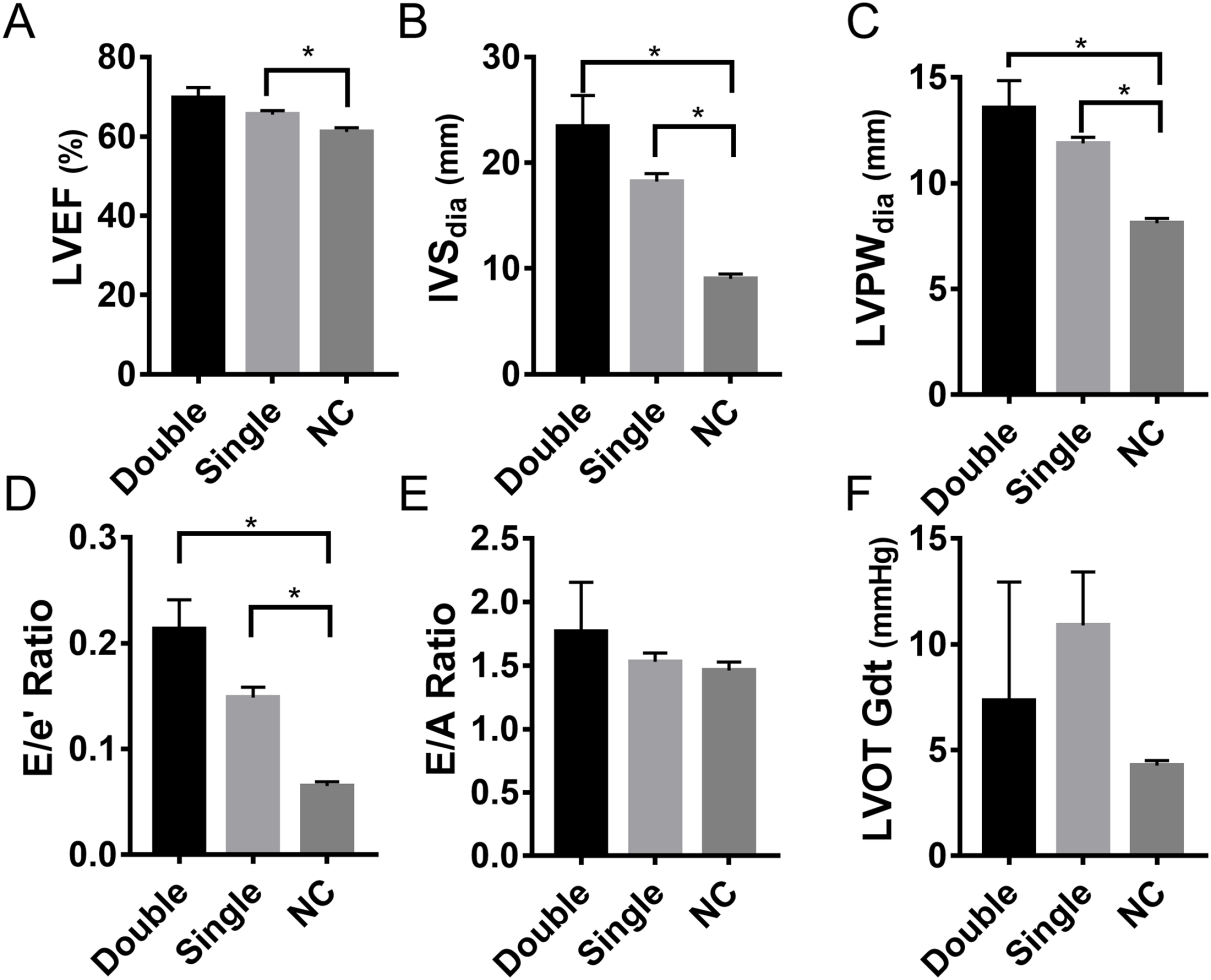
Effect of gene mutation dosage on the pathogenesis of HCM. Comparison of double mutation (Dbl) and single mutation (Sing) with non-carrier (NC) subjects. The severity of pathogenesis between the groups was measured by LVEF, left ventricular ejection fraction (**A**); IVS, interventricular septum thickness (**B**); LVPW, left ventricular posterior wall (free wall) thickness (**C**); E/e’ ratio, ratio of early transmitral flow (E) to left ventricular early diastolic velocity (e’) (**D**); E/A ratio, ratio of early (E) to late (A) ventricular filling velocities (**E**) and LVOT-Gdt, LV outflow tract peak gradient (**F**). * indicates p value of 0.01 or lower by one-way ANOVA analysis.

Double mutations, defined as the presence of one mutation, which is classified as pathogenic, likely pathogenic, or conflicting interpretation of pathogenicity, and a second non-benign mutation, were identified in 10 (6.7%) subjects within the cohort. Nine of the ten subjects exhibited HCM phenotype, but further investigation revealed that the one asymptomatic subject had an *MYH7* mutation that could be characterized as conflicting pathogenicity, while the second *MYBPC3* mutation was defined as benign, resulting in exclusion of the subject from this subgroup. Distinct compound gene mutations, together with their effect on the gene product and likelihood of pathogenicity, are detailed in S4 and S5 Tables. While both single and double mutants exhibited highly significant phenotypic difference compared to control population (Table 5), no phenotypic differences were observed between these two mutant groups (Fig 5A–5F). From these data, it is apparent that compound mutations may substantially increase the risk of developing HCM, without increasing the severity of the HCM phenotype. However, a larger population of patients presenting with compound mutations is required to confirm this.

**Table 5:** Comparison of echocardiographic parameters and ECG findings between patients with single and double mutation in causative genes.

	Double Mutation Group (n = 9)*	Single Mutation Group (n = 105)	*P*-Value
**LVEF, % (mean ± SD)**	69.7 ± 8.1	65.6 ± 9.9	*0*.*40*
**IVS, mm (mean ± SD)**	23.4 ± 8.8	18.24 ± 7.6	0.08
**LVPW, mm (mean ± SD)**	13.6 ± 3.9	11.88 ± 3.0	0.19
**E/ e' Ratio (mean ± SD)**	0.21 ± 0.08	0.15 ± 0.1	0.08
**Decompensated HCM (dHCM), n (%)**‡	0 (0%)	8 (6.8%)	
**Septal reduction, n (%)**§	0 (0%)	5 (4.3%)	
**Normal Sinus Rhythm, n (%)**	5 (55.6%)	50 (47.1%)	
**Paced, n (%)**	2 (22.2%)	12 (11.4%)	
**Bundle branch block, n (%)**	1 (11.1%)	9 (8.6%)	
**R/LVH, n (%)**	1 (11.1%)	19 (18.1%)	
**LAE, n (%)**	0	16 (15.2%)	

Parameters compared include LVEF, left ventricular ejection fraction; IVS interventricular septum; LVPW, left ventricular posterior wall (free wall); E/e’ Ratio, ratio of early transmitral flow (E) to left ventricular early diastolic velocity (e’); ECG findings compared include the number of subjects with normal sinus rhythm, paced rhythm, presence of right or left bundle branch blocks, right or left ventricular hypertrophy and left atrial enlargement.

* Double mutation was defined as one pathogenic, likely pathogenic, or a mutation of conflicting interpretations of pathogenicity, and a second nonbenign mutation in s sarcomeric or non-sarcomeric gene.

^‡^dHCM was defined as LV ejection fraction ≤ 45%

^§^ Septal reduction achieved by either myectomy or alcohol septal ablation. Continuous variables are expressed as mean ± SD, and statistical significance is calculated using unpaired two-tailed Student’s *t*-test.

### Pathogenic missense mutations are location-agnostic

**Fig 6:**
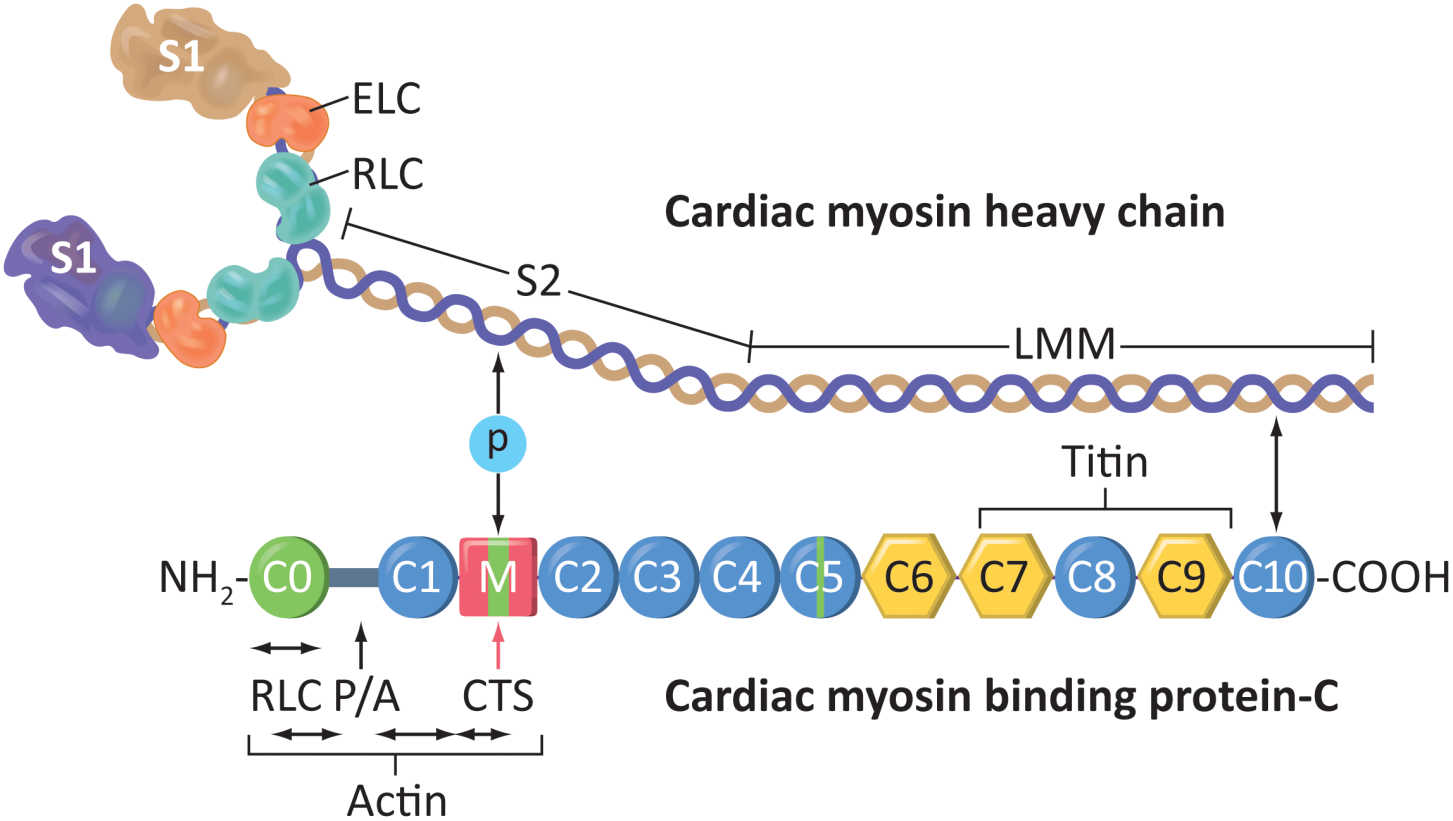
Schematic diagram of cardiac β-myosin heavy chain (β-MHC) and cardiac myosin binding protein-C (cMyBP-C) proteins. cMyBP-C shows the C0-C10 domains and regions of interaction with other proteins, including β-myosin. S1, Myosin heads; ELC, essential light chain; RLC, regulatory light chain; S2, myosin neck region; LMM, light meromyosin. P/A, Proline- and Alanine-rich domain. CTS, Calpain-targeted site.

**Fig 7:**
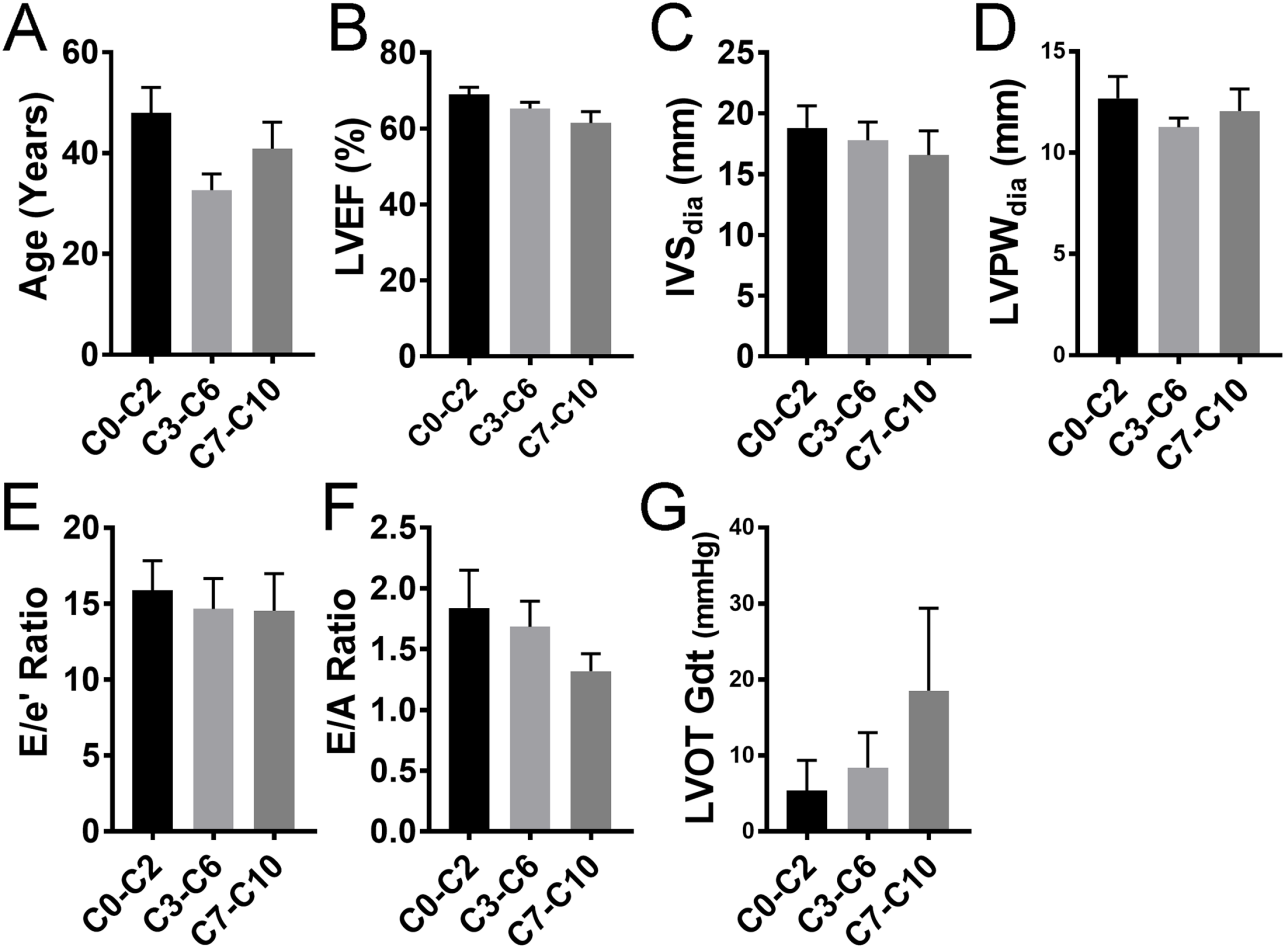
Mutations in cMyBP-C protein interaction domains and their effect on the pathogenesis of HCM. C0-C10 indicates the 11 domains in cMyBP-C protein. The severity of pathogenesis among the groups (N’ C0-C2, C3-C6 and C’ C7-C10 domains) was measured by age at evaluation. (**A**), LVEF, left ventricular ejection fraction (**B**); IVS, interventricular septum thickness (**C**); LVPW, left ventricular posterior wall (free wall) thickness (**D**); E/e’ ratio, ratio of early transmitral flow (E) to left ventricular early diastolic velocity (e’) (**E**); E/A ratio, ratio of early (E) to late (A) ventricular filling velocities (**F**) and LVOT-Gdt, LV outflow tract peak gradient (**G**); n = 14 for C0-C2, n = 19 for C3-C6 and n = 17 for C7-C10.

**Fig 8:**
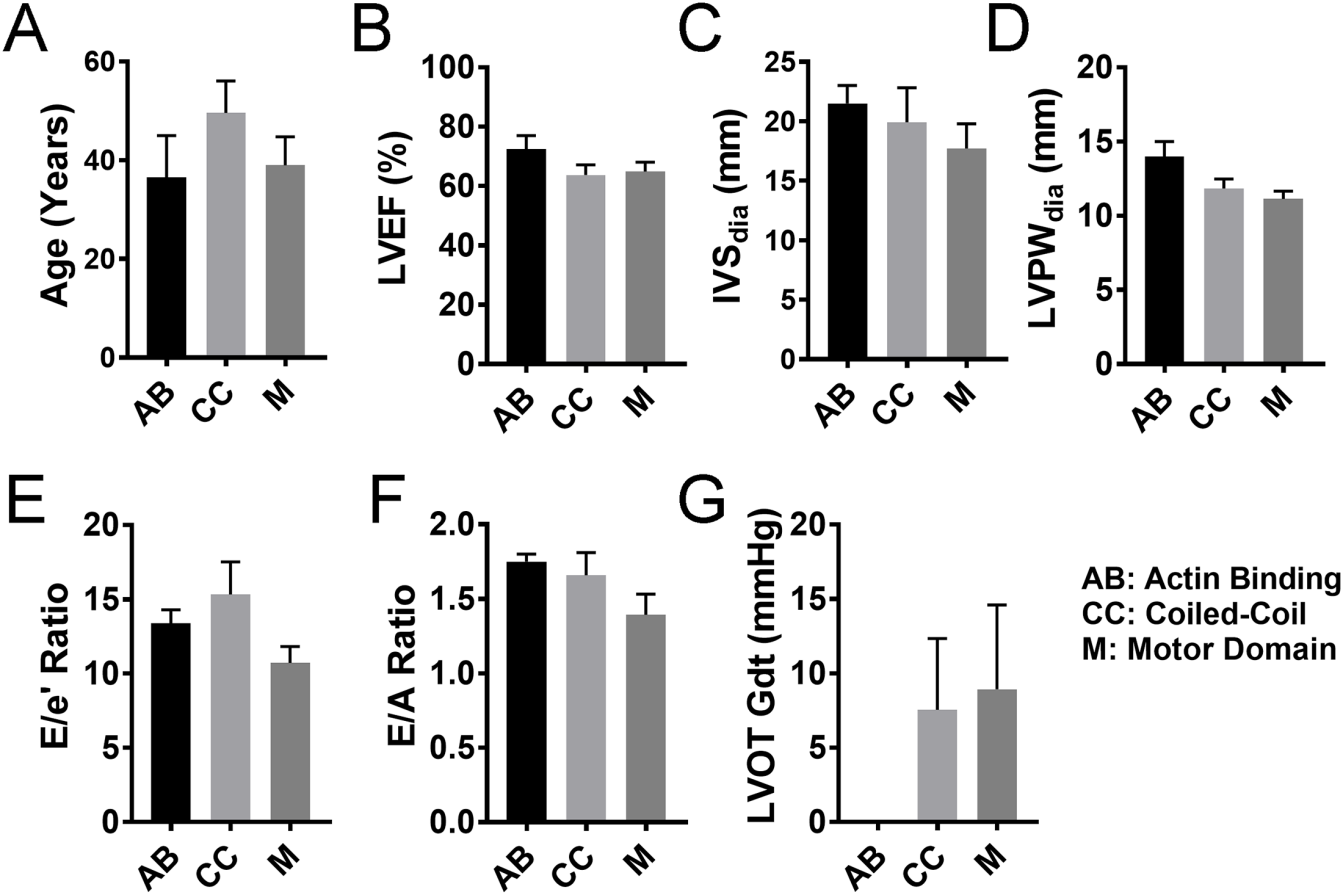
Mutations in β-MHC interaction domains and their effect on the pathogenesis of HCM. The three domains of β-MHC, namely the AB, Actin binding domain; CC, Coiled-coil domain; and M, Motor domain, were compared to each other in order to assess the pathogenicity of mutations that occur in these domains. The severity of pathogenesis among the groups was measured by age at evaluation. (**A**), LVEF, left ventricular ejection fraction (**B**); IVS, interventricular septum thickness (**C**); LVPW, left ventricular posterior wall (free wall) thickness (**D**); E/e’ ratio, ratio of early transmitral flow (E) to left ventricular early diastolic velocity (e’) (**E**); E/A ratio, ratio of early (E) to late (A) ventricular filling velocities (**F**) and LVOT-Gdt, LV outflow tract peak gradient (**G**); n = 4 for AB, n = 13 for CC and n = 13 for M.

*MYBPC3* and *MYH7* encode the sarcomeric proteins cardiac myosin binding-C (cMyBP-C) and β-myosin heavy chain (β-MHC), respectively. As major contractile proteins, both have multiple domain-dependent binding partners. We divided cMyBP-C domains into three functional clusters: C0-C2 that directly interacts with and regulates myosin, C3-C6 that acts as a tether, and C7-C10 that anchors cMyBP-C to the thick filament. Here we compared mutations in these regions of cMyBP-C and the three domains of β-MHC, including motor, actin-binding, and coiled-coil/rod (Fig 6), to echocardiographic outcome. We found no significant association between the site of the mutation and the functional effects of HCM in either cMyBP-C (Fig 7) or in β-MHC (Fig 8). Unfortunately, the small sample size made it difficult to determine domain association in β-MHC.

## Discussion

This study analyzed the interaction of multiple cardiovascular mutations in key causative genes in a cohort of 150 HCM patients, family members who are asymptomatic carriers and non-carriers. The effects of domain-specific mutations and double-dosing mutations in *MHY7* and *MYBPC3* were also investigated. HCM is a unique genetic cardiac disease, which, despite being very common, has a high degree of variability in phenotypic expression and disease penetrance [31]. Even among familial subjects, the variation in phenotypic expressions of the same genotype can be significant [7, 31]. While most studies have identified specific variations that track with the severity of the phenotype [32, 33], the present study looked at all variations seen in the top 18 genes known to cause HCM, and we identified those associated with severe phenotype, irrespective of gender, age and other confounding variables. Among our study subjects, we found that disease penetrance was very high at 69.6% (80 of 115 genotype-positive subjects) and that a high proportion of the familial population (family of probands) presented with an HCM phenotype (38 of 55, 69.0%). This is higher than that previously reported (41% and 15%) [17]. According to Marian and Braunwald [34], over 1,500 mutations have been identified in 26 genes reported to cause HCM. These include nine sarcomeric genes (*ACTC1*, *MYBPC3*, *MYH7*, *MYL2*, *MYL3*, *TNNC1*, *TNNI3*, *TNNT2*, and *TPM1*) and nine non-sarcomere-encoding genes (*CAV3*, *GLA*, *LAMP2*, *MTTG*, *MTTI*, *MTTK*, *MTTQ*, *PRKAG2*, and *TTR*). It was found that more than 94% of HCM subjects expressed mutations in at least one of these genes (34). Interestingly, more than 51.2% of subjects analyzed carried a mutation in *MYBPC3*, while only about 24.8% of patients with HCM carried mutations in *MYH7*. These results stand in contrast to a previous study [35], but they are consistent with similarly sized patient studies, one from a Chinese population [36] and one from a larger Canadian population [37]. While the former study only evaluated *MYBPC3* and *MYH7* [36], the latter study was a meta-analysis that evaluated over 1,400 patients with multiple sarcomeric genes [37].

Mutation analysis of the 18 genes studied in the present work showed that some genetic variations could be identified in both symptomatic carriers and asymptomatic first-degree relatives of mutant carriers. Thus, determining the contribution of these mutations to the phenotype was difficult, as they were likely noncontributing private mutations [9, 38]. Accordingly, such mutations were removed from analysis, and we focused only on gene mutations in that were likely to contribute to the pathogenesis of HCM. Contrary to prevailing evidence that *MYH7* mutations result in a more severe phenotype than *MYBPC3* mutations [17, 35, 39, 40], we found both *MYBPC3* and *MYH7* to be strongly pathogenic with no significant differences in echocardiographic or clinical parameters of patients with mutations in either gene. The relative size of the *MYBPC3* and *MYH7* genes to other HCM-associated sarcomeric genes may increase the probability for disease causing mutations, which could be one of the reasons for the increased frequency of HCM-causing mutations in these two genes [41]. The large gene size (>3000bp) of *MYBPC3* and *MYH7* could be an important factor in the frequency of mutations identified in both these genes relative to other smaller sarcomeric genes (~<1500bp). Owing to increased baseline frequency of mutations, it is also possible that the number of sublethal (pathogenic, but not lethal in pediatric age groups) mutations that slip through the evolutionary filter is also higher compared to diseases like cancer. Among the genes analyzed, *MYBPC3* mutations contributed to more than half of all HCM cases, consistent with previous reports [36, 37]. Interestingly, immediate family members who also carried the variation, but did not have clinically apparent HCM at the time of evaluation, still had increased left ventricular ejection fraction and abnormal diastolic function compared to non-carriers, outcomes suggestive of subclinical HCM [42]

Nine patients with HCM also had double mutations compared to 105 subjects with single mutation. In the present study, a subject was considered to carry a double mutation if 1) one mutation was determined to be pathogenic, likely pathogenic, or have conflicting interpretation of pathogenicity, and 2) a second mutation was determined as non-benign. Importantly, all subjects who presented with double mutation also had a strong cardiomyopathy phenotype [43]. Notwithstanding, comparison of HCM patients with either single or double mutation did not display any significant difference in echocardiographic characteristics, indicating that the presence of a secondary mutation does not significantly alter the pathogenesis of HCM or clinical phenotype when compared to a single mutation. This is to be expected since HCM is a dominant disease such that any failure (single mutation) in the critical path would lead to a phenotype, and acute genetic lesions would be embryonically lethal.

The protein products of *MYBPC3* and *MYH7*, cMyBP-C and β-MHC, are key components of the sarcomere, and both exert their functions through extensive protein-protein interactions. In particular, extensive interactions take place between cMyBP-C and β-MHC. With such close ties, mutations in either of the two genes may significantly alter the structure and function of cardiac muscle, but together, they contribute to >80% of all familial HCM [43]. β-MHC is a globular protein with a motor (M) domain, which includes two actin-binding domains (AB) that directly interact with the actin fiber tracks (thin filaments) and a coiled-coil (CC) domain that intertwines with the CC domain of other myosins. Thus, the M domain allows force transmission on the Z-lines and, in turn, the whole myocyte for contraction (Fig 6). On the other hand, cMyBP-C consists of 11 domains labeled C0 to C10, consisting of immunoglobulin-like or fibronectin-like domains. It crosslinks the thick and thin filaments of the sarcomere and regulates actomyosin interaction, thereby controlling the rate and force of contraction. To achieve this, cMyBP-C can be broadly divided into three functional clusters: C0-C2 that can differentially interact with the myosin motor heads and actin filament, C3-C6 that acts as a neck/tether, and C7-C10 that intertwines with β-myosin coiled-coil tail to anchor in the thick filament (Fig 6). In the present study, we analyzed whether mutations in any of the interacting domains between cMyBP-C and β-MHC could affect the pathogenicity of the HCM-causing mutations. Surprisingly, we did not find any change in phenotypic effects, as reflected by echocardiographic findings, among the various protein domain mutations of cMyBP-C or β-MHC in our study population. Despite multiple protein-protein interactions between cMyBP-C and β-MHC, it is clear from the data that a pathogenic mutation in any location of either protein would result in the failure of the protein as a whole, in turn causing HCM phenotype independent of mutation change in the DNA at any particular locus.

In conclusion, we reported that *MYBPC3* is the most commonly mutated gene among our study population with hypertrophic cardiomyopathy, almost twice that of *MYH7*, which was previously suggested as the most common genetic cause of familial HCM. While it is possible that the higher frequency of *MYBPC3* is specific to our study population, these results are consistent with previous studies from different ethnic groups [36, 37, 44] indicating it is a generalized phenomenon and not based on, or biased by, race or ethnicity. Based on NGS, we evaluated the prevalence, pathogenicity, frequency and location of mutations relative to the clinical phenotype, and we provided strong evidence that *MYBPC3* and *MYH7* can be considered coequal pathogenic causes of familial HCM whose pathogenicity is independent from the location of the mutation on the protein. We also provided clear evidence that compound mutations increase the risk of HCM pathogenesis, and that asymptomatic carriers display subclinical hypertrophic features in terms of wall thickness and left ventricular ejection fraction. Study participants ranged in age from 8 months to 92 years and, moreover, showed strong and consistent HCM phenotype at baseline. Consequently, this study may provide an invaluable platform for the longitudinal evaluation and monitoring of these study subjects who were asymptomatic carriers during the study period.

## Materials & methods

### Study design and population

This study conforms to the principles of the Declaration of Helsinki, and it was approved by the respective Institutional Review Boards. Written informed consent was obtained from all study participants. All HCM patients and their relatives evaluated between July 2010 and August 2016 at the HCM specialized clinic at Aurora St. Luke’s Medical Center, Milwaukee, WI, were included. Normal control subjects, who were non-carriers for gene defects in the 18-gene panel, were recruited throughout the upper Midwest of the United States and were clinically evaluated and imaged at Loyola University Chicago, Maywood, IL. Consenting participants were clinically evaluated, and genetic evaluation was offered for their first-degree relatives and non-carriers. Eighty-two consecutive subjects and 33 of their first-degree relatives were included in this study, together with 35 non-carriers. Subjects were selected based on their diagnosis of HCM, as determined by maximal left ventricular wall thickness of ≥ 15mm, following echocardiographic testing and clinical symptoms, such as chest pain, dyspnea, fatigue, fainting and palpitations. Subjects with secondary factors, such as aortic valve stenosis and myocardial infarction, were excluded from the study. Both Loyola University Chicago and St. Luke’s Medical Center Institutional Review Boards approved the genome-phoneme analyses.

### Electrocardiogram

All participants were tested with 12-lead ECG with rhythm strip to assess for electrical disturbance and potential arrhythmic disorders associated with HCM [45, 46].

### Echocardiography

Cardiac function and morphometry were measured, as described previously by the authors [47]. M-mode and two-dimensional echocardiographic studies were performed from standard transthoracic windows using Acuson Sequoia^™^ (Siemens Medical Solutions, Malvern, PA) and Vivid 7 (GE Healthcare, Waukesha, WI). The echocardiographic equipment was used in accordance with the recommendations of the American Society of Echocardiography. Examinations were performed in a blinded manner by experienced sonographers, and cardiologists interpreted the results [48]. Left ventricular (LV) posterior wall thickness and interventricular septum (IVS) were determined in millimeters using M-mode measurements. Simpson’s method was used to measure left ventricular ejection fraction (LVEF), and diastolic dysfunction was evaluated using E-wave and A-wave diastolic transmitral velocities, E/A ratio, ratio of early transmitral flow (E) to left ventricular early diastolic velocity (e’), E/e’ ratio and deceleration time. These measurements were obtained using spectral pulsed-wave Doppler recordings at the tip of mitral valve leaflets in the apical four-chamber view. Left atrial area and length were measured in apical four-chamber and two-chamber views. Left atrial volume was obtained in orthogonal views and calculated using the following algorithm: 0.85 x Left Atrial Area (Four-Chamber) x Left Atrial Area (Two-Chamber or Apical Long Axis) / (Average of the Two Lengths obtained from Orthogonal Views).

### Genetic testing

DNA sequence analysis of 18 genes associated with HCM was performed at GeneDx (Gaithersburg, MD 20877). Briefly, peripheral blood samples, or oral rinses, were collected from participants, and DNA was isolated from the samples. DNA quantity and quality were checked, and a specific amount of DNA was used to perform targeted gene sequencing of the 18 genes (S1 Table). This was achieved using at least a 100bp paired-end read using the TruSight Cardio sequencing panel kit on Illumina HiSeq sequencers (Illumina, San Diego, CA, USA). Resultant raw DNA reads were checked for quality and trimmed to remove adapter sequences. The sequences were then aligned against the human reference genome build (GRCh37/UCSC hg19), the variations in the sequences collated, reports from GeneDx further analyzed using online resources, e.g., ExAC, dbSNP and HGV, and variant pathogenicity established using the Clinvar database. Among the eighteen cardiomyopathy genes, the presence of variations in multiple genes in an individual was of particular interest. For this analysis, double mutation was defined as the presence of one mutation classified as pathogenic, likely pathogenic, or conflicting interpretation of pathogenicity, and a second non-benign mutation.

### Statistical analysis

Wilcoxon rank-sum tests were used to assess the differences between patient groups for continuous variables, and chi-square test, or Fisher’s exact test, was used for categorical variables. Continuous variables were summarized as mean ± standard error of mean (SEM); discrete variables were described as number and percentage. In order to identify predictors for outcomes, logistic regression analysis was used to calculate odds ratio with 95% CI and to examine the association of each variable with adverse cardiovascular events. All tests were two-tailed, and a P-value <0.05 was considered statistically significant. All statistical analyses were performed with Prism, version 7.4 (GraphPad Inc., La Jolla, CA).

## Supporting information

S1 Table Distribution of familial subjects with respect to disease phenotype. (DOCX)

S2 Table Disease penetrance among the study population. (DOCX)

S3 Table List of previously implicated genes in HCM. (DOCX)

S4 Table List of double mutations identified within the study population. (DOCX)

S5 Table List of all genetic changes analyzed in the study population. (DOCX)
