# Supplementary material for: Hypertrophic cardiomyopathy clinical phenotype is independent of gene mutation and mutation dosage
Source: PLoS One. 2017 Nov 9;12(11):e0187948. doi: 10.1371/journal.pone.0187948 (PMC5679632; doi:10.1371/journal.pone.0187948)
Supplement: S2 Table — (DOCX) [file pone.0187948.s002.docx]

**ONLINE SUPPLEMENTARY: S2 TABLE**

**Hypertrophic cardiomyopathy clinical phenotype is independent of gene mutation and mutation dosage**

Shiv Kumar Viswanathan^1, 2^; Heather K. Sanders^3, 4^; James W. McNamara^1, 2^; Aravindakshan Jagadeesan^2^; Arshad Jahangir^3, 4^; A. Jamil Tajik^3, 4^; Sakthivel Sadayappan^1, 2*^

From the

1. Heart Lung Vascular Institute, Division of Cardiology, Department of Internal Medicine, University of Cincinnati, Cincinnati, OH 45267, USA
2. Department of Cell and Molecular Physiology, Center for Translational Research and Education, Health Sciences Division, Loyola University Chicago, Maywood, IL 60153, USA
3. Aurora Cardiovascular Services, St. Luke’s Medical Center, Milwaukee, WI 53215, USA
4. Center for Integrative Research on Cardiovascular Aging (CIRCA), Aurora Health Care, Milwaukee, WI 53215, USA

**Short title**: *MYBPC3* mutations are predominant in HCM patients

*sadayasl@ucmail.uc.edu

**S2 Table. Penetrance of HCM/HOCM and HREF phenotype among familial and non-familial subjects who carry genetic variations in genes causative of HCM.**

|  | **Probands** | **Family** | **Non-Familial** | **Total** |
| --- | --- | --- | --- | --- |
| Gene Positive | 22 | 33 | 60 | 115 |
| Phenotype Positive | 15 | 23 | 42 | 80 |
| % Penetrance | 68.2 | 69.7 | 70.0 | 69.6 |

Penetrance of obstructive and non-obstructive hypertrophic cardiomyopathy (HOCM/HCM) and HCM with reduced ejection fraction (HREF) phenotypes among familial and non-familial subjects who carried specific cardiomyopathy gene mutations.
