# Supplementary material for: Hypertrophic cardiomyopathy clinical phenotype is independent of gene mutation and mutation dosage
Source: PLoS One. 2017 Nov 9;12(11):e0187948. doi: 10.1371/journal.pone.0187948 (PMC5679632; doi:10.1371/journal.pone.0187948)
Supplement: S3 Table — (DOCX) [file pone.0187948.s003.docx]

**ONLINE SUPPLEMENTARY: S3 TABLE**

**Hypertrophic cardiomyopathy clinical phenotype is independent of gene mutation and mutation dosage**

Shiv Kumar Viswanathan^1, 2^; Heather K. Sanders^3, 4^; James W. McNamara^1, 2^; Aravindakshan Jagadeesan^2^; Arshad Jahangir^3, 4^; A. Jamil Tajik^3, 4^; Sakthivel Sadayappan^1, 2*^

From the

1. Heart Lung Vascular Institute, Division of Cardiology, Department of Internal Medicine, University of Cincinnati, Cincinnati, OH 45267, USA
2. Department of Cell and Molecular Physiology, Center for Translational Research and Education, Health Sciences Division, Loyola University Chicago, Maywood, IL 60153, USA
3. Aurora Cardiovascular Services, St. Luke’s Medical Center, Milwaukee, WI 53215, USA
4. Center for Integrative Research on Cardiovascular Aging (CIRCA), Aurora Health Care, Milwaukee, WI 53215, USA

**Short title**: *MYBPC3* mutations are predominant in HCM patients

*sadayasl@ucmail.uc.edu

**S3 Table. Eighteen genes previously shown to carry mutations that cause cardiomyopathy.**

| **Sarcomeric Genes** | **Non-Sarcomeric Genes** |
| --- | --- |
| ACTC1 | CAV1 |
| MYBPC3 | GLA |
| MYH7 | LAMP2 |
| MYL2 | MTTG |
| MYL3 | MTTI |
| TNNC1 | MTTK |
| TNNI3 | MTTQ |
| TNNT2 | PRKG2 |
| TPM1 | TTR |

Cardiomyopathy causative genes represented in the GeneDx HCM Diagnostic test panel and can be sub classified as sarcomeric and non-sarcomeric genes based on the compartment of their respective protein localization.
