# Supplementary material for: Hypertrophic cardiomyopathy clinical phenotype is independent of gene mutation and mutation dosage
Source: PLoS One. 2017 Nov 9;12(11):e0187948. doi: 10.1371/journal.pone.0187948 (PMC5679632; doi:10.1371/journal.pone.0187948)
Supplement: S4 Table — (DOCX) [file pone.0187948.s004.docx]

**ONLINE SUPPLEMENTARY: S4 TABLE**

**Hypertrophic cardiomyopathy clinical phenotype is independent of gene mutation and mutation dosage**

Shiv Kumar Viswanathan^1, 2^; Heather K. Sanders^3, 4^; James W. McNamara^1, 2^; Aravindakshan Jagadeesan^2^; Arshad Jahangir^3, 4^; A. Jamil Tajik^3, 4^; Sakthivel Sadayappan^1, 2*^

From the

1. Heart Lung Vascular Institute, Division of Cardiology, Department of Internal Medicine, University of Cincinnati, Cincinnati, OH 45267, USA
2. Department of Cell and Molecular Physiology, Center for Translational Research and Education, Health Sciences Division, Loyola University Chicago, Maywood, IL 60153, USA
3. Aurora Cardiovascular Services, St. Luke’s Medical Center, Milwaukee, WI 53215, USA
4. Center for Integrative Research on Cardiovascular Aging (CIRCA), Aurora Health Care, Milwaukee, WI 53215, USA

**Short title**: *MYBPC3* mutations are predominant in HCM patients

*sadayasl@ucmail.uc.edu

**S4 Table. Hypertrophic cardiomyopathy patients with double mutations**

| **Gene 1** | **Nucleotide Change** | **AA Change** | **Clinvar Interpretation** | **Gene 2** | **Nucleotide Change** | **AA Change** | **Clinvar Interpretation** |
| --- | --- | --- | --- | --- | --- | --- | --- |
| **MYBPC3** | c.1504C>T | p.Arg502Trp | Conflicting interpretations of pathogenicity | **MYBPC3** | c.3682C>T | p.Arg1228Cys | Conflicting interpretations of pathogenicity |
| **MYBPC3** | c.2373dupG | p.Trp792ValfsX41 | Novel (Frame shift, Truncation) | **MYBPC3** | c.2170C>T | p.Arg724Trp | Conflicting interpretations of pathogenicity |
| **MYBPC3** | c.442G>A | p.Gly148Arg | Uncertain significance | **MYBPC3** | c.1433C>T | p.Ser478Leu | Uncertain significance |
| **MYH7** | c.5326A>G | p.Ser1776Gly | Conflicting interpretations of pathogenicity | **MYBPC3** | c.76A>G | p.Ser236Gly | Benign/Likely benign |
| **PRKAG2** | c.667T>A | p.Tyr223Asn | Uncertain significance | **LAMP2** | c.26_-15dup12 | NA |  |
| **MYH7** | c.1988G>A | p.Arg663His | Pathogenic/ Likely pathogenic | **PRKAG2** | c.1304A>G | p.Asn435Ser | Uncertain significance |
| **TNNT2** | c.832C>T | p.Arg278Cys | Unknown, Damaging by SIFT | **TTR** | c.280G>C | p.Asp94His | Uncertain significance |

All subjects with double mutation had phenotypic HCM. Table details the first and second mutation in causative genes, amino acid change and their consequence as reported on ClinVar database. Novel mutation’s effect was predicted using SIFT and PolyPhen databases.
