# Supplementary material for: Hypertrophic cardiomyopathy clinical phenotype is independent of gene mutation and mutation dosage
Source: PLoS One. 2017 Nov 9;12(11):e0187948. doi: 10.1371/journal.pone.0187948 (PMC5679632; doi:10.1371/journal.pone.0187948)
Supplement: S5 Table — (DOCX) [file pone.0187948.s005.docx]

**ONLINE SUPPLEMENTARY: S5 TABLE**

**Hypertrophic cardiomyopathy clinical phenotype is independent of gene mutation and mutation dosage**

Shiv Kumar Viswanathan^1, 2^; Heather K. Sanders^3, 4^; James W. McNamara^1, 2^; Aravindakshan Jagadeesan^2^; Arshad Jahangir^3, 4^; A. Jamil Tajik^3, 4^; Sakthivel Sadayappan^1, 2*^

From the

1. Heart Lung Vascular Institute, Division of Cardiology, Department of Internal Medicine, University of Cincinnati, Cincinnati, OH 45267, USA
2. Department of Cell and Molecular Physiology, Center for Translational Research and Education, Health Sciences Division, Loyola University Chicago, Maywood, IL 60153, USA
3. Aurora Cardiovascular Services, St. Luke’s Medical Center, Milwaukee, WI 53215, USA
4. Center for Integrative Research on Cardiovascular Aging (CIRCA), Aurora Health Care, Milwaukee, WI 53215, USA

**Short title**: *MYBPC3* mutations are predominant in HCM patients

*sadayasl@ucmail.uc.edu

**S5 Table. List of all genetic changes analyzed in the study population.**

| **GENE** | **Nucleotide Change** | **Family ID** | **Participant ID** |
| --- | --- | --- | --- |
| ACTC1 | c.268 C>T | 25 | PT-51 |
|  |  |  | PT-52 |
| GLA | c.616 C>G | #N/A | PT-107 |
|  | c.713 G>A | 23 | PT-47 |
|  |  |  | PT-48 |
|  |  | #N/A | PT-66 |
| JPH2 | c.661 T>C | #N/A | PT-103 |
| LAMP2 | c.864+2 T>C | #N/A | PT-61 |
|  | c.26_-15dup12 | #N/A | PT-108* |
| LMNA | c.1190 G>A | #N/A | PT-113* |
| MYBPC3 | c.1028delC | #N/A | PT-56 |
|  | c.1235 123delITT | 13 | PT-25 |
|  |  |  | PT-26 |
|  | c.1390+4C>T | #N/A | PT-58* |
|  | c.1433 C>T | 9 | PT-18* |
|  | c.1457 G>A | 11 | PT-22 |
|  |  |  | PT-23 |
|  |  | #N/A | PT-60 |
|  | c.1458-6 G>A | 1 | PT-1 |
|  |  |  | PT-2 |
|  |  |  | PT-3 |
|  |  |  | PT-4 |
|  | c.1471 G>A | #N/A | PT-64 |
|  | c.1484 G>A | 14 | PT-27 |
|  |  |  | PT-28 |
|  |  |  | PT-29 |
|  | c.1504 C>T | 7 | PT-16* |
|  |  | #N/A | PT-67 |
|  | c.1624 G>C | #N/A | PT-68 |
|  | c.1669 G>A | 4 | PT-10 |
|  |  |  | PT-11 |
|  |  |  | PT-12 |
|  |  |  | PT-13 |
|  |  |  | PT-9 |
|  | c.2170 C>T | #N/A | PT-70* |
|  | c.2373dupG | #N/A | PT-70* |
|  | c.2450 G>A | #N/A | PT-74 |
|  | c.2455_2459delATGCG | #N/A | PT-78 |
|  | c.2541 C>G | #N/A | PT-79 |
|  | c.2771 C>T | #N/A | PT-81 |
|  | c.2864_2865delCT | 6 | PT-15 |
|  | c.2870 C>G | #N/A | PT-82 |
|  | c.3089 T>C | #N/A | PT-83 |
| MYBPC3 | c.3098 G>A | #N/A | PT-84 |
|  | c.3106 C>T | #N/A | PT-85 |
|  | c.3190+2 T>G | #N/A | PT-86 |
|  | c.3192dupC | 3 | PT-7 |
|  |  |  | PT-8 |
|  | c.330+2 T>G | #N/A | PT-88 |
|  | c.3305 T>A | #N/A | PT-89 |
|  | c.3330+5 G>C | #N/A | PT-91 |
|  | c.3490+1 G>T | 5 | PT-14 |
|  | c.3682 C>T | 7 | PT-17 |
|  |  |  | PT-16* |
|  | c.3697 C>T | 10 | PT-20 |
|  |  |  | PT-21 |
|  |  | 12 | PT-24 |
|  | c.3776delA | #N/A | PT-95 |
|  | c.3791 G>T | #N/A | PT-96 |
|  | c.442 G>A | 9 | PT-18* |
|  |  |  | PT-19 |
|  | c.472 G>A | #N/A | PT-100 |
|  |  | #N/A | PT-98 |
|  | c.50 G>A | #N/A | PT-102 |
|  | c.532 G>A | 2 | PT-5 |
|  |  |  | PT-6 |
|  | c.649 A>G | #N/A | PT-105 |
|  |  | #N/A | PT-106 |
|  | c.76 A>G | #N/A | PT-110* |
|  | c.821+1 G>A | #N/A | PT-109 |
|  |  | #N/A | PT-112 |
|  | c.927-9 G>A | #N/A | PT-113* |
|  | c653 A>G | #N/A | PT-115 |
| MYH7 | c.1231 G>A | #N/A | PT-57 |
|  | c.1360 A>G | #N/A | PT-59 |
|  | c.1370 T>C | #N/A | PT-62 |
|  | c.1491 G>T | 18 | PT-35 |
|  |  |  | PT-36 |
|  |  |  | PT-37 |
|  | c.1615 A>G | 17 | PT-33 |
|  |  |  | PT-34 |
|  | c.1988 G>A | 22 | PT-45* |
|  |  |  | PT-46* |
|  |  | #N/A | PT-69 |
|  |  | #N/A | PT-75 |
|  | c.2334 C>G | #N/A | PT-76 |
|  | c.2546 T>C | #N/A | PT-80 |
|  | c.2606 G>A | 15 | PT-30 |
|  | c.2761 G>A | 16 | PT-31 |
|  |  |  | PT-32 |
|  | c.2782 G>A | #N/A | PT-92 |
|  | c.3749 G>C | 20 | PT-41 |
| MYH7 | c.4159 G>A | #N/A | PT-97 |
|  | c.4199 C>G | #N/A | PT-99 |
|  | c.4353+5 G>A | #N/A | PT-101 |
|  | c.4399 C>G | #N/A | PT-104 |
|  | c.5317 C>A | 21 | PT-42 |
|  |  |  | PT-43 |
|  |  |  | PT-44 |
|  | c.5326 A>G | #N/A | PT-110* |
|  | c.563 C>A | #N/A | PT-111 |
|  | c.727 C>T | 19 | PT-38 |
|  |  |  | PT-39 |
|  |  |  | PT-40 |
| MYL3 | c.170 C>G | 26 | PT-53 |
|  |  |  | PT-54 |
|  |  |  | PT-55 |
| PRKAG2 | c.667 T>A | #N/A | PT-108* |
|  | c.1304 A>G | 22 | PT-45* |
|  |  |  | PT-46* |
| TNNC1 | c.469 A>C | #N/A | PT-94 |
| TNNI3 | c.421 C>T | #N/A | PT-63 |
| TNNT2 | c.257 A>C | #N/A | PT-73 |
|  | c.514 G>T | #N/A | PT-90 |
|  | c.732 G>T | #N/A | PT-87 |
|  | c.832 C>T | #N/A | PT-114 |
|  |  | #N/A | PT-71 |
|  |  | #N/A | PT-77* |
|  | c.857 G>A | #N/A | PT-65 |
|  |  |  | PT-93 |
| TPM1 | c.253 G>A | 24 | PT-49 |
|  |  |  | PT-50 |
|  | c.644 C>T | #N/A | PT-72 |
| TTR | c.14 G>A | #N/A | PT-58* |
|  | c.280 G>C | #N/A | PT-77* |

A list of all nucleotide changes detected in all participants in the study. Identical mutations are grouped, and related subjects harboring the same genetic mutation are indicated. #N/A indicates that subjects family members were not screened in this study. All nucleotide changes are represented using standard sequence variation nomenclature (<http://varnomen.hgvs.org/>) and individuals with compound mutations are marked with an asterisk (*).
